# Assessment of asymmetry and trajectory during repeated twenty-meter sprints in court sports wheelchair athletes

**DOI:** 10.3389/fspor.2025.1511167

**Published:** 2025-03-28

**Authors:** Florian Brassart, Sadate Bakatchina, Ilona Alberca, Zoé Pomarat, Eric Watelain, Thierry Weissland, Arnaud Faupin

**Affiliations:** ^1^ Pôle P3R—UFAM, Hôpitaux Paris-Est Val-de-Marne, Saint-Maurice, Val-de-Marne, France; ^2^ J-AP2S - No. 201723207F, Université de Toulon, La Garde, Var, France; ^3^ UMR 5218, PMH_Dysco, Université de Bordeaux, Pessac, Gironde, France

**Keywords:** steering, asymmetry, wheelchair sports, sprints, fatigability

## Abstract

**Introduction:**

Manual wheelchair (MWC) propulsion relies on upper limb power, coordination, and endurance. Propulsion asymmetry can reduce efficiency, yet the impact of fatigability on upper limb asymmetry remains underexplored. This study aimed to compare propulsion performance and asymmetry between wheelchair basketball (WB) and wheelchair rugby (WR) players and assess the effect of fatigability on asymmetry during repeated sprints.

**Method:**

13 WB and 10 WR players from French national teams performed 6 × 20 m sprints with 20-second recovery intervals. Inertial measurement units (IMUs) were placed on wheel spokes and the trunk captured wheel velocity and trunk motion. The Instantaneous Symmetry Index (ISI) quantified propulsion asymmetry.

**Results and discussion:**

Both groups showed performance decline across sprints, with WB players experiencing a drop in maximal power output and WR players showing reduced average sprint velocity. Asymmetry was highest at sprint initiation, with WB players exhibiting greater ISI values than WR players. Interestingly, WR players demonstrated reduced asymmetry at sprint onset, possibly due to sport-specific anthropometric adaptations. Trunk motion remained stable over sprints but was more pronounced in WB players.

**Conclusion:**

The results highlight distinct fatigue-related adaptations in propulsion asymmetry between WB and WR players. The study's findings underscore the need for further exploration into the nuanced dynamics of propulsion and asymmetry in parasport performance.

## Introduction

Manual wheelchair (MWC) propulsion is an intricate biomechanical activity that demands a harmonized interplay of strength, coordination, and endurance from the upper limbs. Asymmetry in propulsion patterns, particularly between the dominant and non-dominant sides, can significantly influence the efficiency and safety of MWC users, potentially leading to long-term musculoskeletal complications (1, 2). Prior investigations have underscored the variability of interlimb asymmetry during MWC propulsion on roller ergometers, noting disparities in parameters such as distance traveled between wheels, maximum attained speed, and power exerted on the wheel rim (2, 3). Moreover, the magnitude of asymmetry has been shown to be modulated by factors like task difficulty, rolling resistance, and terrain inconsistencies (4, 5).

Goosey-Tolfrey et al. (3) suggested that functional capacities can impact asymmetry and power generation, as they observed greater asymmetry in the high-point group of wheelchair rugby (WR) players (3). In a previous study, the authors observed that performance differences between wheelchair basketball (WB) and WR players are primarily dictated by their level of impairment and upper limb function. WB players, who generally have higher functional capacities, tend to generate greater power output than WR players (6). Additionally, strapping height and abdominal capacity vary according to functional classification, influencing trunk mobility and its contribution to propulsion.

In the literature, it has been observed that during straight-line movement with sport manual wheelchairs, propulsion asymmetry can influence steering, resulting in lateral oscillations of the MWC (7). This can lead to augmented trunk movements, subsequently increasing $\dot{V}$ O_2_ and heart rate (8, 9). In sports predominantly involving the lower limbs, fatigability has manifested varied effects on asymmetry (10–15). However, its ramifications on upper limb asymmetry, especially in the context of manual wheelchair propulsion, warrant further exploration. The effect of fatigability on the progression of these steering movements remains unexamined.

In this study, leveraging the precision of inertial measurement units (IMUs), we aimed to compare propulsion performance and asymmetry during repeated sprint tests between male athletes engaged in wheelchair basketball and those in wheelchair rugby. The second aim was to examine the evolution of upper limb asymmetry in relation to accumulated fatigability during these repeated sprints.

Given the distinct biomechanical demands of each sport, we hypothesize that WB and WR players will exhibit differential propulsion asymmetry and performance patterns. Furthermore, based on the literature on lower limb sports, we postulate that fatigability will progressively influence propulsion asymmetry during repeated sprints.

## Method

### Participants

A total of 13 male WB players and 10 male WR players from French national teams participated in this study (see Table 1 for participant characteristics). Participants trained four days a week and competed in matches on weekends. Inclusion criteria were the use of a manual wheelchair for sports and the absence of current back or upper limb pain or injury. Participants’ pathologies included paraplegia (*n* = 7), incomplete tetraplegia (*n* = 5), lower limb amputation (*n* = 1), congenital malformation (agenesis) (*n* = 3), spina bifida (*n* = 1), sequelae of poliomyelitis (*n* = 1), neuropathy (*n* = 1) cerebral palsy (*n* = 2), arthrogryposis (*n* = 1) and hip dysplasia (*n* = 1). Exclusion criteria included athletes who exhibited pain during the warm-up, untreated injuries, or were undergoing treatment for viral or bacterial illnesses.

**Table 1:** Participant characteristics.

Participants characteristics	Wheelchair basketball (*n* = 13)	Wheelchair rugby (*n* = 10)	All participants (*n* = 23)
Body mass (kg)	71 (10)	73 (12)	72 (11)
Age (yrs)	29 (7)	33 (7)	31 (7)
Training age (yrs)	12 (5)	8 (4)	10 (5)
LP/HP	7/6	5/5	12/11
Pathology distribution and classification	Paraplegia (*n* = 6, classes 1–3)	Paraplegia (*n* = 1, class 3)	
	Arthrogryposis (*n* = 1, class 2.5)	Tetraplegia (*n* = 5, classes 0.5–2.5)	
	Dysplasia (*n* = 1, class 4.5)	Neuropathy (*n* = 1, class 3)	
	Cerebral palsy (*n* = 1, class 3)	Cerebral palsy (*n* = 1, class 2)	
	Agenesis (*n* = 1, class 4)	Agenesis (*n* = 2, classes 1.5–3.5)	
	Spina bifida (*n* = 1, class 1.5)		
	Amputee (*n* = 1, class 3.5)		
	Sequelae of poliomyelitis (*n* = 1, class 3)		

This table presents the characteristics of the participants, divided into two groups: Wheelchair basketball (WB) and Wheelchair rugby (WR). The data are presented as mean (standard deviation). LP refers to the “low point” classification (WB: 1–2.5 and WR: 0.5–1.5), and HP refers to the “high point” classification (WB: 3–4.5 and WR: 2–3.5).

### Materials

In this study, Inertial Measurement Units (IMU) were used to evaluate sprint performances (128 Hz, Bluetooth module, Wheelperf System, Atounovation, Versailles, France). Four IMUs were affixed to the athlete and their wheelchair, one on each rear wheel (with the Z-axis of the gyrometer perpendicular to the wheel axis) as described in literature (16–20), one on the frame, and one on the participant's trunk (positioned between vertebrae T2 and T8).

### Protocols

The six repeated sprint tests were conducted on a wooden surface, with 20-second recovery intervals between each sprint. The countdown for the recovery period began as soon as the participants crossed the finish line. Wheelchair basketball and wheelchair rugby players performed the test in their personal sports wheelchairs, using their own support systems, including strapping, seat height and depth, and customized backrest height. The tyre pressure was the same as that used in training or competition, ranging between 8 and 9 bar. The warm-up entailed 5 min of maneuvering the wheelchair in circular patterns around the gymnasium, complemented by brief accelerations. Data on the rear wheel diameter, participant weight, and wheelchair weight were documented. The 20 m sprint's commencement and conclusion were distinctly demarcated, with the experimenters initiating the sprint using a countdown. This protocol was chosen because it effectively induces significant fatigue in manual wheelchair athletes (21).

Six sprints were performed. To assess the influence of asymmetry on fatigability, the first and the sixth sprints were compared. To evaluate the influence of asymmetry on performance, the fastest (ST) and slowest (LT) sprints were identified and analyzed, regardless of their order within the series.

### Data processing

To minimize wheel slip, the 20-meter track was cleaned to reduce dust accumulation, and athletes’ tires were wiped before each trial to optimize traction. Although an IMU was placed on the wheelchair frame, its acceleration readings were affected by noise, drift, and frame vibrations, making velocity estimation through numerical integration unreliable. Therefore, we used the directly measured angular velocity from the IMUs attached to the MWC wheels to ensure greater accuracy. Consequently, the linear speed of the MWC was computed by multiplying the rotational speeds of the wheels around their axis of rotation, as captured by the gyrometer of the IMUs, with the wheel's radius. It's important to note that sports MWC incorporate a camber angle to enhance stability and maneuverability. This angle affects the wheel rotation measurement during chair pivoting, as the sensor registers both the wheel's rotational speed and the chair's rotational speed around its vertical axis. To account for this potential measurement discrepancy, we used the method introduced by Pansiot et al. (18) and refined by Fuss (17) to ascertain the authentic rotational speed of the wheel (17, 18). The start and end of the sprints were detected using IMU data: the start was defined as the moment when the average speed of both wheels exceeded 0.06 m/s, while the end was determined when the wheelchair had traveled 20 meters from the start, calculated by integrating the average speed of both wheels.

The force developed by players during the sprint ($F_{IMU}$) was computed with the second law of Newton as Equation 1.

(1) $$F_{IMU}=m_{t}*acc\;+\;F_{rr}+F_{aero}$$

Here $m_{t}$ was the system's total mass consisting of the participant and their wheelchair, ***acc*** was the linear acceleration, $F_{rr}$ was the rolling resistance force, and $F_{aero}$ was the aerodynamic drag force. To obtain the acceleration (acc), we followed a similar approach to Nagahara et al. (22), where velocity data were fitted using a 4th-degree polynomial (pV) at each point in × as equation 2. The argument ***p*** is a vector of length *n* + 1 whose elements was the coefficients (in descending powers) of a 4th-degree polynomial. This method reduces the impact of intra-stroke variations and provides a reliable representation of acceleration dynamics, aligning with established sprint modeling techniques (22, 23).

To calculate acceleration (acc), we followed a similar approach to Nagahara et al. (22), where velocity data were fitted using a 4th-degree polynomial (pV) at each point in x. smoothes the data and reduces the impact of intra-stroke variations. This method provides a reliable representation of acceleration dynamics, aligning with established sprint modeling techniques.

(2) $$pV(x)=p1x^{4}+p2x^{3}+p3x^{2}+p4x+p5$$

The coefficients for the polynomial (*p1, p2…, p5)* were returned to be the best fit (in a least-squares sense) for the velocity data $\left(V_{T}\right)$. The coefficients and the polynomial were computed with the MATLAB functions “polyfit” and “polyval”. $F_{rr}$ was estimated using a deceleration test (24) and computed with equation 3. The deceleration test was performed over a 5 m section in the middle of a 20 m track. The MWC was initially pushed by an experimenter to reach a speed of approximately 7–10 km/h, ensuring straight-line motion. After the push, the wheelchair was allowed to decelerate naturally over 5 m before being briefly stopped by the experimenter. The test was conducted in both directions, and the final deceleration value was obtained by averaging the two trials. The deceleration phase was defined as the interval between 0.5 s after the end of the push and 0.5 s before the experimenter stopped the wheelchair. IMU data from the wheels were used to determine this deceleration. While this method is based on Sauret et al. (24), we acknowledge that weight distribution affects drag force (25, 26), making this approach an estimation rather than an exact calculation (25, 26). In the equation 3, g was the gravitational constant, $\mu_{r}$ was the rolling resistance coefficient compute as the deceleration value divided by g. $k_{f}$ was the coefficient of influence of speed on friction (27).

(3) $$F_{rr}=m_{t}g(\mu_{r}+k_{f}pV^{2})$$

The aerodynamic drag force was obtained with equation 4 where $C_{d}A$ was the drag coefficient in the function of the frontal area of the system (28), while the frontal area (A) was estimated based on a standard athlete's position during propulsion using a frontal photograph. ρ was the air density (1.22 kg.m^−3^).

(4) $$F_{aero}=\frac{1}{2}\rho C_{d}{ApV}^{2}$$

**Figure 1:**
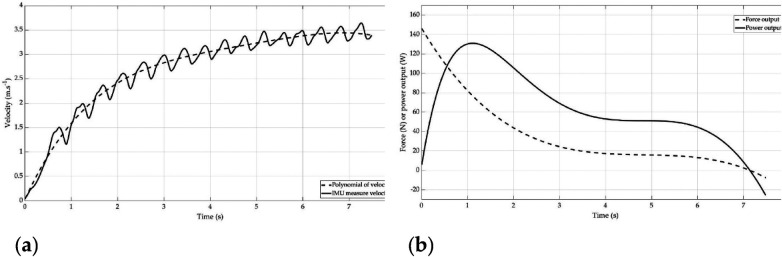
Linear velocity of a manual wheelchair basketball player measured (solid line) and polynomial of this velocity (dotted line) **(a)** power (solid line) and force (dotted line) calculated from acceleration, speed and mass of the same participant **(b)**.

The power output estimation (PIMU) was computed with the multiplication between the force (FIMU) and the velocity (***pV***) (Figure 1b). For each sprint, the force-velocity (F-V) profile was made with each point of FIMU accordingly to each point of pV.

### Asymmetries

**Figure 2:**
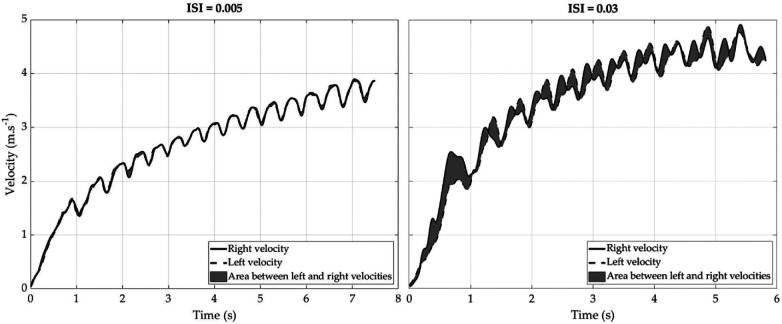
Effect of right and left velocity symmetry on the Instantaneous Symmetry Index (ISI) during a 20 m sprint in wheelchair with inertial measurement unites.

The Instantaneous Symmetry Index (ISI) was calculated according to the method proposed by Chénier et al. (29). The ISI was defined as the absolute area between the right and the left side curves of the assessed variable, normalized by the sum of the absolute areas under both curves (Figure 2).

(8) $$ISI=\;\frac{\int_{t1}^{t2}\;|R-L|dt}{\int_{t1}^{t2}\;|R|dt+\int_{t1}^{t2}\;|L|dt}$$

Where R and L was the assessed variable on the right and left sides, respectively, and where ***t1*** and ***t2*** represented the start and the end of the period during which the ***ISI*** was calculated.

We used velocity data to calculate the ISI because this measure is considered more representative of the participant's action than other metrics. We calculated the ISI at three-time points during the sprint: at the start of the sprint (corresponding to the first three cycles of propulsion), in the middle of the sprint (corresponding to the cycles of propulsion between the start and the end), and at the end of the sprint (corresponding to the last five cycles of propulsion). The ISI is representative of steering movement by the computation of accumulation of lateral displacements during propulsion, defined by the velocity differences between wheels.

### Trunk kinematic

To assess the influence of the trunk on MWC maneuverability, trunk kinematics were monitored using an IMU securely positioned on each athlete's back with a strap, between the T2 and T8 vertebrae. Due to space constraints between the athlete and the wheelchair seat, no IMU was placed on the pelvis. Additionally, relative angular measurements between the trunk and the wheelchair frame could not be obtained, as the IMU data from the frame exhibited a high noise level.

Given these limitations, the Trunk Range of Motion (TRM) was chosen as the most relevant parameter to quantify trunk movement. TRM represents the amplitude of trunk motion in the anteroposterior axis relative to the global earth coordinate system. It was measured as the difference between the maximum and minimum trunk flexion during each propulsion cycle, and the average between cycles was used for the comparison. Specifically, the angular velocity of trunk inclination was measured, and the trunk flexion angle was derived from rotation matrices based on quaternions provided by the WheelPerf IMUs, following a y-z-x sequence (19, 30).

Athletes were instructed to remain still in their initial position before the effort. In this phase, the inclination angle of the IMU was visually assessed by the experimenter. The measured offset was then subtracted from the computed Euler angles to correct for initial misalignment.

### Statistics

Analysis was conducted using R (31), RStudio (Integrated Development Environment for R. Posit Software, PBC, Boston, MA. URL http://www.posit.co/) with “corrplot” package (https://github.com/taiyun/corrplot), “car” package (https://r-forge.r-project.org/projects/car/) and “multcomp” package (http://multcomp.R-forge.R-project.org). The normal distribution of the data was tested with the Shapiro–Wilk test. The homoscedasticity of the data was calculated with Levene's test. A two-way ANOVA was proposed to examine the interaction between sports and sprints. For non-normally distributed data, a non-parametric alternative, the Kruskal–Wallis test, was used. After the ANOVA, *post-hoc* tests with Bonferroni correction were employed to make pairwise comparisons between groups while controlling for the family-wise error rate. A Linear Mixed-Effects Model was used to analyze the evolution of variables across different sprints for both WR and WB players. The model accounted for repeated measures within participants and provided insights into both fixed effects (effects of sprints) and random effects (variability across participants).

## Results

The two-way ANOVA (group × time) demonstrated superior performance for WB players compared to WR in terms of force, power, maximal, and mean velocity, all of which contribute to a shorter sprint time. Between sprint n°1 and n°6, there is a noticeable decline in performance. These differences were statistically significant for both WB and WR in relation to maximal, and mean velocity. Only the WR group showed a significant difference in sprint time between the two sprints. And WB showed a significant difference in power (Table 2). Between the ST and the LT sprint, there are significant differences for WB and WR in maximal and mean velocity and in sprint time, but no significant difference in asymmetry (Table 3).

**Table 2:** Comparison between wheelchair basketball and wheelchair rugby players and between the first (1) and the last (6) sprint of the repeated sprint test.

Variables	Wheelchair basketball		Wheelchair rugby		*p*
	Sprint *n*°1	Sprint *n*°6	Sprint *n*°1	Sprint *n*°6	
Maximal force (*N*)	301.8 (74.2)	286 (25.9)	226 (63.8)	224.1 (66.3)	**
Maximal velocity (m.s^−1^)	5.39 (0.49)	5.24 (0.39)	4.43 (0.71)	4.21 (0.72)	**$££
Maximum power (W)	413.7 (127)	371.3 (28.9)	247.6 (105.9)	237.9 (104.6)	**$
Mean velocity (m.s^−1^)	3.68 (0.31)	3.63 (0.24)	3.1 (0.46)	3 (0.55)	**$$££
Mean velocity start (m.s^−1^)	2.11 (0.14)	2.04 (0.26)	1.73 (0.26)	1.73 (0.37)	**
Sprint time (s)	5.49 (0.49)	5.56 (0.37)	6.6 (0.99)	6.84 (1.39)	**££
ISI_start	0.03 (0.013)	0.032 (0.02)	0.028 (0.009)	0.019 (0.007)	*
ISI_middle	0.014 (0.004)	0.016 (0.012)	0.012 (0.007)	0.012 (0.003)	
ISI_end	0.015 (0.008)	0.015 (0.008)	0.01 (0.005)	0.01 (0.005)	
ISI	0.018 (0.005)	0.019 (0.009)	0.014 (0.006)	0.012 (0.005)	
TRM start (°)	26.1 (6.4)	27.1 (6.1)	15 (11.9)	17.9 (21.5)	*
TRM middle (°)	16.4 (2.9)	18 (2.3)	10.5 (7.5)	13.2 (17.2)	*
TRM end (°)	17.5 (4.1)	16.5 (2.4)	9.7 (6.8)	11.6 (11.8)	**

Data are mean (standard deviation). The Instantaneous Symmetry Index (ISI) and The Range of Motion of the trunk (TRM) are compute during the start, middle and the end part of the sprint.

* and ** represent significant differences between wheelchair basketball and wheelchair rugby players (*p* < 0.05 and *p* < 0.001 respectively).

$ and $$ represent significant differences between the first and the last sprints for wheelchair basketball players (*p* < 0.05 and *p* < 0.001 respectively).

£ and ££ represent significant differences between the first and the last sprints for wheelchair rugby players (*p* < 0.05 and *p* < 0.001 respectively).

**Table 3:** Comparison between wheelchair basketball and wheelchair rugby players and between the shortest (ST) and longest timed (LT) sprint of the repeated sprint test.

Variables	Wheelchair basketball		Wheelchair rugby		*p*
	ST	LT	ST	LT	
Sprint number	3 (2)	4 (2)	1 (0)	5 (1)	
Maximal Force (*N*)	284.3 (71)	259.2 (46.1)	226 (63.8)	218.2 (66.3)	**
Maximal Velocity (m.s^−1^)	5.39 (0.54)	5.29 (0.38)	4.43 (0.71)	4.01 (0.72)	**$££
Maximum Power (W)	378.2 (94.4)	377.4 (101.3)	247.6 (105.9)	219 (104.6)	**
Mean velocity (m.s^−1^)	3.72 (0.3)	3.53 (0.24)	3.1 (0.46)	2.84 (0.55)	**$$££
Mean velocity start (m.s^−1^)	2.12 (0.15)	2.04 (0.23)	1.73 (0.26)	1.64 (0.27)	**$$£
Sprint Time (s)	5.43 (0.48)	5.71 (0.38)	6.6 (0.99)	7.25 (1.39)	**$$££
ISI_start	0.036 (0.014)	0.035 (0.018)	0.028 (0.009)	0.02 (0.007)	*
ISI_middle	0.012 (0.005)	0.019 (0.01)	0.012 (0.007)	0.014 (0.003)	
ISI_end	0.013 (0.008)	0.019 (0.007)	0.010 (0.005)	0.011 (0.005)	*
ISI	0.016 (0.006)	0.022 (0.008)	0.014 (0.006)	0.014 (0.005)	**
TRM start (°)	23.8 (8.2)	25.9 (4.7)	15 (11.9)	21.2 (21.5)	*
TRM middle (°)	19.6 (3.3)	17.9 (2.4)	10.5 (7.5)	15.8 (17.2)	*
TRM end (°)	18 (4.2)	16.6 (3.2)	9.7 (6.8)	13.1 (11.8)	**

Data are mean (standard deviation). The Instantaneous Symmetry Index (ISI) and The Range of Motion of the trunk (TRM) are computed during the start, middle and the end part of the sprint.

* and ** represent significant differences between wheelchair basketball and wheelchair rugby players (*p* < 0.05 and *p* < 0.001 respectively).

$ and $$ represent significant differences between ST and LT sprints for wheelchair basketball players (*p* < 0.05 and *p* < 0.001 respectively).

£ and ££ represent significant differences between ST and LT sprints for wheelchair rugby players (*p* < 0.05 and *p* < 0.001 respectively).

No significant difference exists in the TRM between sprints. However, WB athletes exhibit greater TRM during propulsion compared to WR. The TRM is more pronounced at the beginning of the sprint and diminishes as the sprint progresses.

Higher ISI values are observed at the start of sprint for all groups when compared to the mid-point and end of each sprint. WB athletes tend to display greater asymmetry than WR. This difference is statistically significant on average during the starts of sprints, at both the first and last sprints. There is no difference of ISI between sprints.

It was observed that the fastest sprints were not always the first sprints for wheelchair basketball players. Among the 13 players, only 3 had their best sprint as the first one, 3 had their best on the second, 3 on the third, 2 on the fourth, and 1 on the sixth. In contrast, for wheelchair rugby players, the fastest sprint was consistently the first one (Table 3).

**Figure 3:**
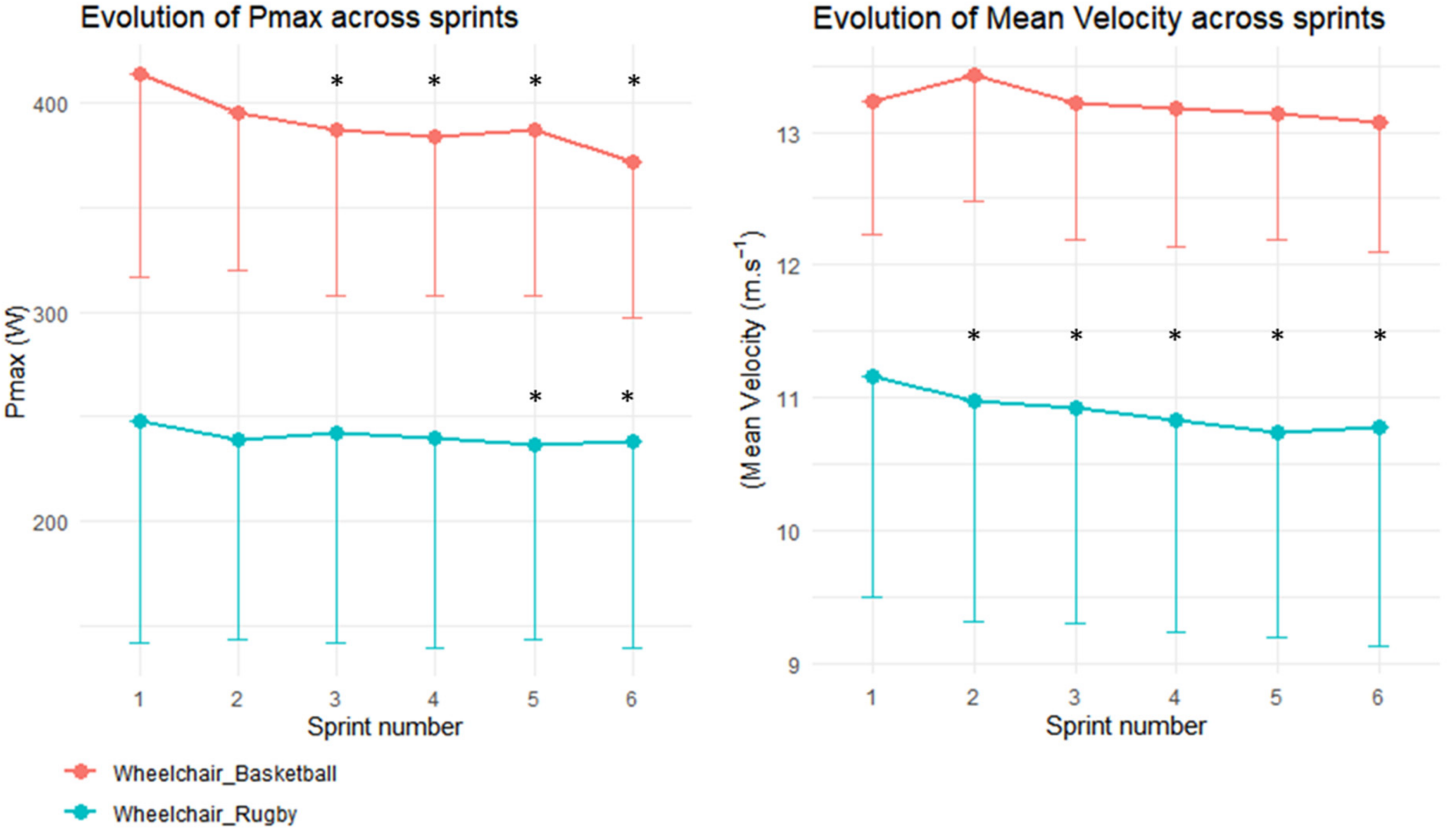
Evolution of maximal power output (pmax) and mean velocity across sprints. Red dots and lines represent wheelchair basketball players end blue dots and lines represent wheelchair rugby players. * represents significant difference of the sprint performance with the first sprint (*p* < 0.05) for the corresponding group directly below it.

For WR, the Linear Mixed-Effects Model revealed significant differences in Pmax between sprint 5 and sprint 1, as well as between sprint 6 and sprint 1. Additionally, there was a significant reduction in average velocity for all sprints when compared to the first one. In contrast, for WB, significant differences in Pmax were observed between sprints 3, 4, 5, and 6 relative to sprint 1, with no significant variation in average velocity (as depicted in Figure 3).

**Figure 4:**
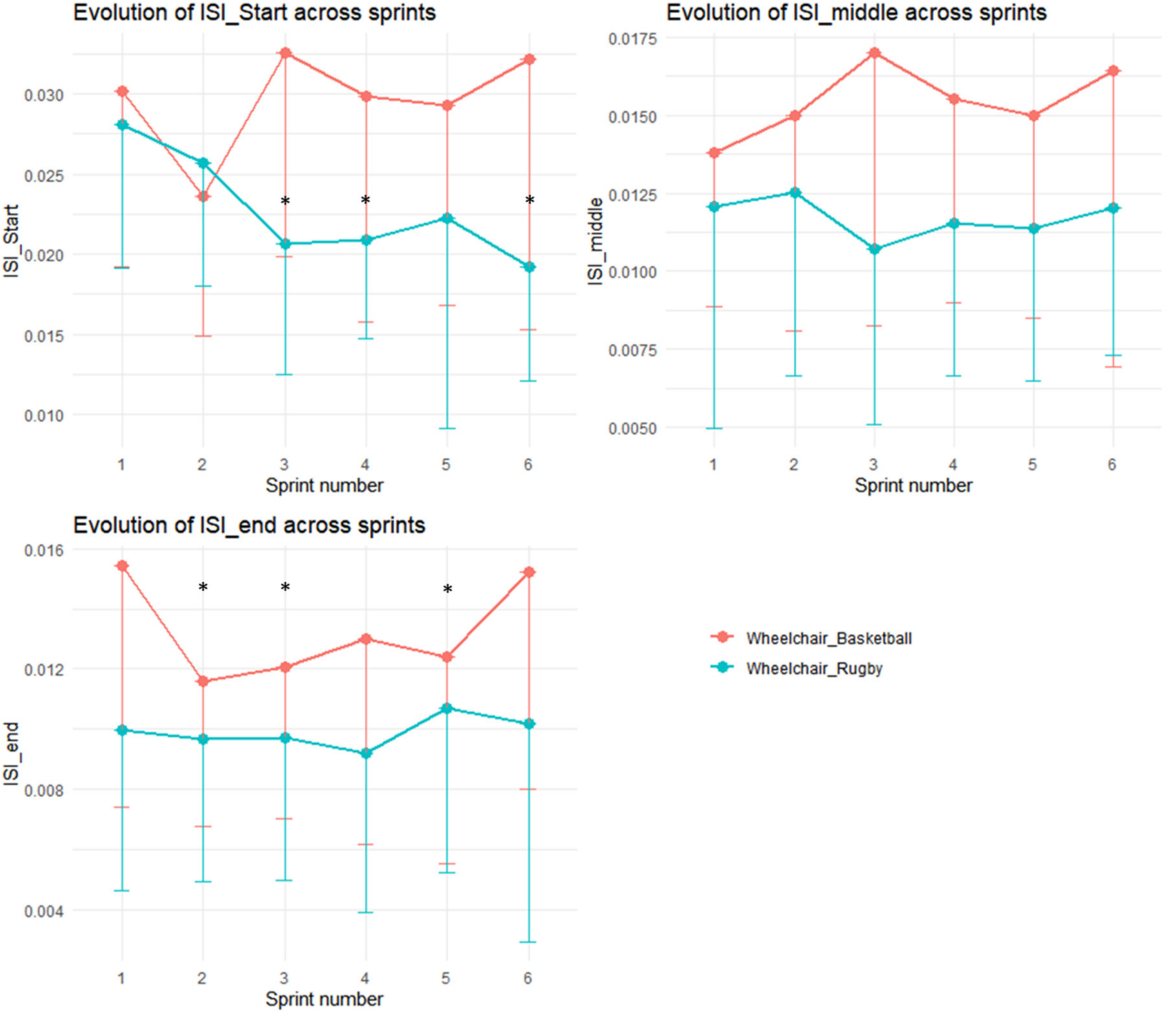
Evolution Instantaneous Symmetry Index (ISI), at the start (ISI_start), the middle (ISI_middle) and at the end (ISI_end) of each sprint, across sprints. Red dots and lines represent wheelchair basketball players end blue dots and lines represent wheelchair rugby players. * represents significant difference of the sprint performance with the first sprint (*p* < 0.05) for the corresponding group directly below it.

Regarding the ISI value, a repeated measures ANOVA revealed a significant difference between the first and second sprints for WB, but no such difference was observed for WR. For WR players, the ISI at the beginning of each sprint decreased, with significant deviations from the first sprint noted for sprints 3, 4, and 6. The ISI values at the end of each sprint varied for WB players, and significant differences were observed for sprints 2, 3, and 5 when compared to sprint 1. However, there were no significant differences in the ISI values measured at the midpoint of each sprint, as illustrated in Figure 4.

## Discussion

Our primary results highlight that asymmetry, measured by ISI, remained consistent throughout the test for all players. This suggests that despite the performance decline observed during repeated sprints, asymmetry did not significantly change, even in the presence of fatigability. Both ISI and TRM were notably more pronounced at the start of each sprint, with initial asymmetries appearing to be more prominent. These findings imply that the level of asymmetry, while present, may not be the primary factor driving the decrease in performance. Furthermore, the superior performance of WB players compared to WR players was observed, and both groups experienced a decrease in performance and longer sprint times as the test progressed, although the sprint time significantly increased only for WR players.

The main distinctions between WB and WR players lie in the level of impairment and the upper limb disabilities stemming from their respective pathologies. Additionally, some WR players exhibit anthropometrical asymmetries in the upper limbs. The superior performances noted for WB players in this study align with the findings of Goosey-Tolfrey and Leicht (6). Their review of the literature revealed that WB and high-classification players tend to be more powerful than their low-classification and WR counterparts (6). In this study, both WB and WR players demonstrated divergent performance patterns in response to fatigability. For WB players, the manifestation of fatigability was evident in the decrease of maximal power output throughout consecutive sprints. Conversely, WR players exhibited fatigability through a decline in average sprint velocity between successive sprints. Notably, these findings underscore the variations in fatigability adaptations, contingent upon the specific sport and the players’ unique impairments. Another illuminating observation is that, on average, WB players recorded their shortest sprint time during the third sprint and the longest during the fourth. This suggests that WB players modulate their effort to maintain a consistent velocity in the face of increasing fatigability.

Regarding the TRM, no changes were observed across repeated sprints. At the onset of the sprint, the range of motion was, on average, greater than during the sprint's continuation. Furthermore, WB players with greater trunk mobility exhibited more pronounced movements. This can also explain better performances observed in WB players (32).

In this investigation, the chosen index of symmetry quantified the deviation during straight-line MWC movement. A higher ISI value indicates increased steering. The ISI was elevated, implying more pronounced velocity variations between sides, at the beginning of the sprint compared to its end. This might be influenced by the trunk motion and the heightened force and power demands relative to the remainder of the sprint. The diminishing asymmetry at the start of the sprint, exclusively observed in WR players, remains noteworthy. However, as start velocity does not significantly decline between the first and sixth sprints, there is no clear fatigue effect on initial acceleration capacities. Despite this, asymmetry still decreases, suggesting that other mechanisms may be at play. The relatively low value of ISI in this test can be partially explained by the necessity for the players to maintain symmetry in order to move straight.

### Limitations

A notable limitation of this study, beyond the modest sample size of elite wheelchair basketball and rugby players, is the significant variability inherent within the wheelchair athletes population, leading to a notably diverse and heterogeneous sample. Additionally, recruiting high-level WB and WR players is challenging due to their broad geographical distribution and the subsequent difficulty in accessing specialized laboratory-based measurement systems. As a result, securing large cohorts of elite athletes is especially arduous within this demographic. Regarding the methodology, the use of instrumented wheels with force sensors on the handrims could have provided additional relevant information for a proper evaluation of asymmetry on the field. Furthermore, it is possible that the chosen protocol did not induce enough fatigue to observe significant adjustments in asymmetry, despite the notable decline in performance. This suggests that future studies should explore different protocols to induce a more substantial level of fatigue and better capture its effects on asymmetry.

### Perspectives

A future direction for this study would be to employ a roller ergometer to further investigate force and velocity asymmetries in a controlled environment and compare these findings with on-court data. This would allow us to better understand certain biomechanical and physiological mechanisms. Additionally, it would provide insight into possible adaptations to asymmetry (or lack thereof) in a population with varied pathologies, in response to fatigue during high-intensity efforts and in a performance context. However, given the limitations of ergometers in replicating sport-specific demands, future research could also explore more ecologically valid approaches. Instrumented wheels, despite their current constraints in terms of weight, ergonomics, and cost, could offer valuable insights into real-world compensatory mechanisms. Additionally, refining the fatigue protocol to induce a greater level of fatigue, while incorporating turn tests, could provide a more accurate assessment of how fatigue impacts maneuverability and propulsion asymmetry in sport-specific settings.

## Conclusion

To conclude, this study primarily aimed to investigate the relationship between upper limb asymmetry, fatigability, and performance during propulsion. Our findings indicate that asymmetry did not significantly influence performance, nor did fatigability impact asymmetry or steering movements. It is possible that our protocol did not induce enough fatigue in the athletes, and therefore, alternative protocols should be explored to address this question in field studies. Despite this, we observed a subtle reduction in starting asymmetry among wheelchair rugby (WR) players. This suggests that there may be specific motor coordination adjustments in a population with greater functional limitations at the trunk and upper limbs. Additionally, wheelchair basketball (WB) players demonstrated superior performance compared to WR players, with WB players experiencing a decline in maximal propulsion power output as fatigability set in, whereas WR players showed a reduction in average velocity capacities. Lastly, our investigation into the range of motion of the trunk did not reveal a significant link to either propulsion asymmetry or fatigability, further refining the understanding of these factors in wheelchair sports.

## Data Availability

The raw data supporting the conclusions of this article will be made available by the authors, without undue reservation.
